# Trace Zr Addition Enhances Strength and Plasticity in Cu-Zr/Al_2_Cu/Al Alloys via Local FCC-to-BCC Transition: Molecular Dynamics Insights on Interface-Specific Deformation and Strain Rate Effects

**DOI:** 10.3390/ma18071480

**Published:** 2025-03-26

**Authors:** Shuang Li, Wenyan Wang, Yunfeng Cui, Jingpei Xie, Aiqin Wang, Zhiping Mao, Feiyang Zhang

**Affiliations:** 1 School of Materials Science and Engineering, Henan University of Science and Technology, Luoyang 471023, China; shuangbright_3230@126.com (S.L.); cuiyf1996@163.com (Y.C.); jingpeixie@163.com (J.X.); aiqin_wang888@163.com (A.W.); zpmao@haust.edu.cn (Z.M.); zhangfy2714603621@163.com (F.Z.); 2 Digital Molding Engineering Research Center of Tungsten and Molybdenum Materials in Henan Province, Luoyang 471822, China; 3 Provincial and Ministerial Co-Construction of Collaborative Innovation Center for Non-Ferrous Metal New Materials and Advanced Processing Technology, Henan University of Science and Technology, Luoyang 471023, China

**Keywords:** Cu-Zr/Al_2_Cu/Al system, molecular dynamics simulations, mechanical properties, interfaces, phase transformation, dislocation, strain rate

## Abstract

This study investigates how Zr doping influences the deformation behavior of Cu-Zr/Al_2_Cu/Al composites through molecular dynamics simulations. The impact of Zr content (ranging from 0 to 0.8 wt%) and strain rate on phase evolution, dislocation dynamics, and fracture mechanisms under vertical and horizontal tensile loading was examined. The results indicate that Zr doping achieves a balance between strength and plasticity by means of solute drag, amorphization, and phase competition. At a Zr concentration of 0.2 wt%, the formation of the body-centered cubic (BCC) phase reached a peak (22.04% at ε = 0.11), resulting in a maximum tensile strength of 9.369 GPa while maintaining plasticity due to limited face-centered cubic (FCC) decomposition. A moderate Zr content of 0.6 wt% maximizes strength through amorphization but significantly diminishes plasticity due to excessive FCC-to-BCC transitions. Higher Zr concentrations (0.8 wt%) lead to solute supersaturation, which suppresses phase transitions and slightly reduces toughness by causing hexagonal close-packed (HCP) phase accumulation. The strain rate markedly enhances both strength and plasticity in vertical loading by accelerating dislocation interactions. Vertical tensile deformation initiates brittle fracture, whereas horizontal loading results in ductile failure through sequential load transfer from Al_2_Cu layers to Al/Cu interfaces, ultimately causing interfacial decohesion. These findings underscore the essential roles of Zr content and strain rate in modulating phase transformations and interface responses. The research offers a framework for creating gradient Zr-doped or multi-scale composites with optimized strength, plasticity, and damage tolerance suitable for aerospace and electronics applications, where trace Zr additions can reinforce Cu matrices.

## 1. Introduction

Copper–aluminum laminated composite materials represent a novel class of materials fabricated through a specialized composite process, enabling the attainment of robust metallurgical bonding at the interface between dissimilar base metals possessing distinct physical, chemical, and mechanical properties. These materials exhibit high levels of electrical and thermal conductivity comparable to that of pure copper while simultaneously achieving a weight reduction of 40% and being priced at merely 60% of the cost of pure copper. Consequently, significant research has been undertaken to explore the fabrication techniques and performance characteristics of single-layer composites comprising pure copper and pure aluminum [1,2]. Applications of copper–aluminum laminated composites include copper-clad aluminum wires, copper–aluminum plates and strips, and copper–aluminum joint materials, which are widely used in aerospace, electric power, transportation, and related industries [3,4,5,6].

Despite their advantages, copper–aluminum laminated composite materials are prone to cracking and fracturing at the interface during service, leading to overall material failure [7,8,9,10]. The microstructural characteristics of the interface, including phase composition and distribution, exert a significant influence on the bonding strength and energy, rendering the Cu/Al interface a subject of intense investigation among scholars worldwide in recent years [11,12,13]. Higher bonding strength at the interface and a smaller strength differential between Cu and Al enhance the cooperative deformation capability of these composites [14]. The introduction of alloying elements can modify the crystal structure, dislocation density, and defect distribution in the material, consequently affecting its strength, hardness, fracture toughness, and other mechanical properties [15,16,17,18,19,20,21]. The incorporation of trace amounts of Zr into Cu not only has a negligible impact on the base metal’s conductivity but also serves as an effective precipitation-strengthening agent with excellent solid solution effects [22,23,24,25,26,27]. We used a horizontal double-roll casting and rolling apparatus to conduct experiments on C18150 copper plates and 1060 industrial pure aluminum with dimensions of 400 mm × 200 mm × 2.5 mm. The resulting copper/aluminum/copper laminated composite material exhibited outstanding tensile strength and elongation, reaching 201 MPa and 16%, respectively, coupled with excellent conductivity. The electrical conductivity of the copper side was approximately 87% IACS, satisfying the requirements for high-strength and high-conductivity copper–aluminum composite materials.

Research on atomic-scale deformation processes in Cu-Zr/Al laminated composites is still in its early stages, and the lack of clarity regarding the correlation between microstructure and material deformation mechanisms hinders further enhancement and optimization of the toughening performance of these composites. Thus, there is a pressing need for advanced and convenient research methodologies to delve into the fundamental nature of deformation in Cu-Zr/Al laminated composite materials at a more microscopic level and to analyze their deformation mechanisms. Leveraging molecular dynamics simulations, it is possible to replicate the organizational structure and strain evolution of the material during elastic deformation, plastic deformation, and fracture at the microscopic scale. This approach can provide insights into the deformation and fracture behavior at the interface of the laminated heterogeneous structure while also reducing experimental cycle times and costs [28,29].

This study presents a constructed Cu-Zr/Al_2_Cu/Al interface model and employs molecular dynamics simulations to investigate the vertical and parallel interface tensile deformation behaviors under varying Zr doping levels. The objective was to determine the optimal process. The study also delves into the impact of different strain rates on the mechanical properties of Cu-Zr/Al_2_Cu/Al, focusing on atomic migration, dislocation motion, and phase structural evolution during deformation. Additionally, it examines how Zr content influences stress–strain characteristics and strengthening mechanisms, providing insights into the microscopic deformation mechanisms of Cu-Zr/Al_2_Cu/Al composites.

## 2. Models and Methods

**Figure 1:**
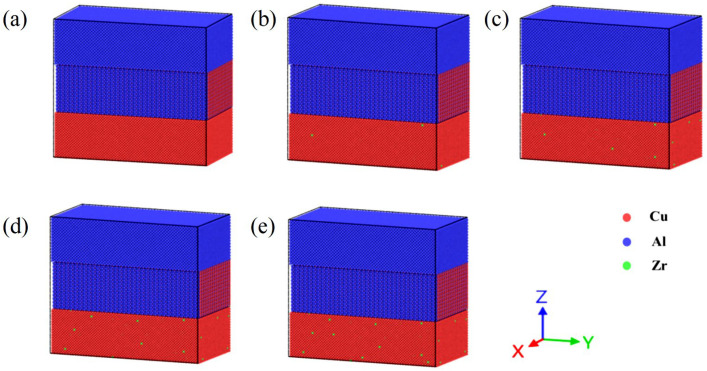
Cu-Zr/Al_2_Cu/Al model doped with different contents of Zr. (**a**) Cu-Zr/Al_2_Cu/Al; (**b**) Cu-0.2Zr/Al_2_Cu/Al; (**c**) Cu-0.4Zr/Al_2_Cu/Al; (**d**) Cu-0.6Zr/Al_2_Cu/Al; (**e**) Cu-0.8Zr/Al_2_Cu/Al.

The initial atomic structure model was constructed using the Atomsk modeling software (Atomsk, Version beta-0.13.1), developed by Pierre Hirel (Unité Matériaux et Transformations, Université Lille 1, Villeneuve d’Ascq, France), as illustrated in Figure 1a. Periodic boundary conditions are implemented in the x and y directions, while the z-axis features one fixed end and the other exhibiting a contractive boundary. The topmost and bottommost layers along the z-axis are designated as rigid and fixed layers of Cu atoms, with a thickness of four atomic layers, respectively. The central region sequentially comprises a Cu single-crystal structure, an Al_2_Cu single-crystal structure, and an Al single-crystal structure from bottom to top, with the contact surface oriented along the ideal (001) plane. The model dimensions are defined as 29a_1_ × 29a_1_ × 28a_1_, 17a_2_ × 17a_2_ × 8b_2_, 26a_3_ × 26a_3_ × 25a_3_, and 29a_1_ × 29a_1_ × 2a_1_. Here, a_1_ (a_1_ = 0.3615 nm) denotes the lattice constant of Cu, while a_2_ (a_2_ = 0.608 nm) and b_2_ (b_2_ = 0.482 nm) represent the lattice constants of Al_2_Cu. The lattice constant of Al is denoted as a_3_ (a_3_ = 0.4050 nm).

In the lower copper layer, 0.2%, 0.4%, 0.6%, and 0.8% of copper atoms are randomly substituted with Zr, in accordance with the target weight percentage, with the spatial distribution of Zr adhering to a Poisson distribution. This methodology simulates the solid-state alloying process while effectively preventing artificial aggregation. Given the structural instability observed in actual simulations, along with notable pre-stress and atomic potential energy, it is crucial to relax each segment of the model. This relaxation is accomplished by conducting the simulation at 300 K for 50 ps, followed by the integration of the segments, as depicted in Figure 1b–e. In this integration mode, the parameters concerning the coordinate axis orientation and dimensions for Cu, Al_2_Cu, and Al are detailed in Table 1. Specifically, the x-axis, y-axis, and z-axis of Al_2_Cu, Al, and Cu correspond to the crystal directions of [100], [010], and [001], respectively. The final interface model measures 20.22 nm × 10.47 nm × 18.75 nm and comprises a total of 275,948 atoms. The trajectory of the atoms adheres to Newton’s laws of motion. The motion equations are numerically integrated using the Verlet method. The initial velocities of the atoms are determined by the Maxwell speed distribution, with a time step set to 1 fs.

The simulations conducted in this study utilized the LAMMPS software package (LAMMPS 64-bit, https://rpm.lammps.org/windows/legacy/admin/64bit/index.html (accessed on 15 June 2023)), developed by Sandia National Laboratories (Albuquerque, NM, USA). The analytical embedded atomic method (EAM) potential proposed by Cai et al. served as the potential function for this research [30]. This atomic potential has been extensively employed in studies investigating atomic diffusion behavior in metals and intermetallic compounds. The results are largely consistent with Miedema’s alloy theory and first-principle calculations. The simulation process involved applying tensile deformation along the z- or x-axis direction, while maintaining the temperature at 300K.

## 3. Results and Discussion

### 3.1. Tensile Deformation at Cu-Zr/Al_2_Cu/Al Vertical Interfaces

#### 3.1.1. Influence of Different Zr Content on Tensile Deformation at Cu/Al_2_Cu/Al Vertical Interfaces

**Figure 2:**
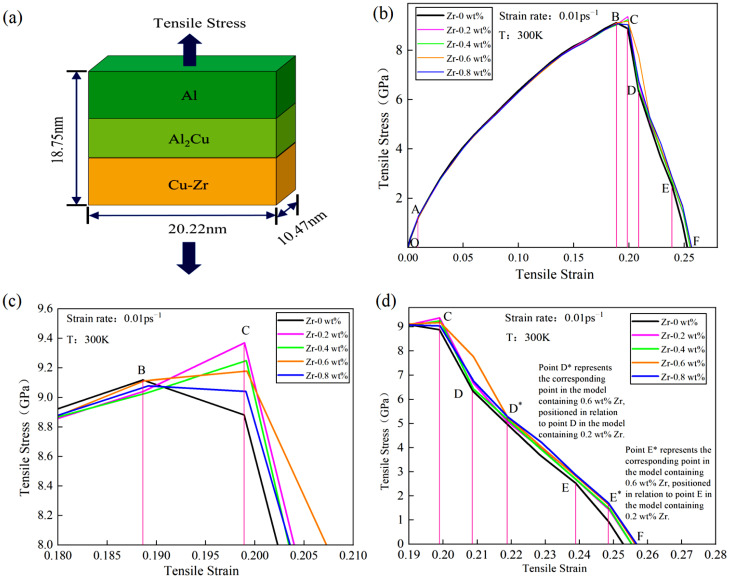
Tensile model and stress–strain curve of Cu-Zr/Al_2_Cu/Al vertical interface with different Zr content: (**a**) Cu-Zr/Al_2_Cu/Al vertical interface tensile model; (**b**) tensile stress–strain curve of Cu-Zr/Al_2_Cu/Al vertical interface; (**c**) local enlarged view of the plastic deformation stage; (**d**) local enlarged view of the tensile fracture stage.

Under a temperature condition of 300 K, tensile simulations for the five models depicted in Figure 1 were conducted. The tensile direction, illustrated in Figure 2a, is perpendicular to the interface model, with a tensile strain rate of 0.01 ps^−1^. The stress–strain curves obtained from the Cu-Zr/Al_2_Cu/Al models, which were doped with varying Zr contents and subjected to tensile deformation, are presented in Figure 2b. It is evident that all five models underwent similar deformation processes under tensile loading: the OA section represents the elastic deformation stage, the AC section denotes the plastic deformation stage, and the CF section corresponds to the necking fracture stage. During the elastic stage (segment OA), all models exhibit a linear increase in the stress–strain curve, with the slope (elastic modulus E) showing minimal dependence on the Zr doping level. This observation indicates that Zr doping does not significantly alter the elastic modulus. Furthermore, it suggests that during the elastic stage, the interface model is predominantly influenced by the matrix materials (Al/Cu), and the solid solution or interface effects of Zr are insufficient to induce notable changes in the lattice parameters.

With the conclusion of the elastic deformation stage, the material progressively transitions into the plastic deformation stage under continuous tensile loading. The variations in the stress–strain curves of models with different Zr doping contents during the plastic deformation segment AB are not significant. However, in the local deformation segment BC, models with different doping levels exhibit distinct trends. As illustrated in Figure 2c, models doped with 0.2 wt%, 0.4 wt%, 0.6 wt%, and 0.8 wt% Zr all demonstrate ultimate tensile strengths surpassing that of the undoped model. The order of strength is as follows: σ0 wt% < σ0.8 wt% < σ0.6 wt% < σ0.4 wt% < σ0.2 wt%. Detailed information is presented in Table 2. Notably, when Zr content is at 0.2 wt%, the ultimate tensile strength of the model reaches a maximum of 9.369 GPa. As Zr content increases from 0.2 wt% to 0.6 wt%, the BC segment exhibits a “peak-rising” trend; however, this peak gradually diminishes with further increases in Zr content. At 0.8 wt% Zr, the BC segment displays a “peak-declining” trend, indicating that the value at point C is lower than that at point B. This suggests that surface Zr doping significantly enhances the ultimate tensile strength of the material. Within a certain range, the improvement effect of Zr doping initially increases and then decreases with higher doping levels. Particularly, at 0.2 wt% Zr, the material achieves optimal strength. Furthermore, the changes in the BC segment of the stress–strain curve indicate that an appropriate amount of Zr doping (0.2 wt%–0.6 wt%) is beneficial for maintaining good mechanical properties during the local deformation stage and prolongs the material’s fracture process. Conversely, when Zr content is excessively high (e.g., 0.8 wt%), although initial strength is enhanced, the material’s bearing capacity decreases in the later stages of local deformation. Excessive Zr doping may introduce factors that hinder the plastic deformation of the material, such as grain boundary embrittlement or stress concentration. Therefore, in the design of materials with surface Zr doping, it is crucial to control the doping amount appropriately to optimize the material properties.

During the CF necking fracture stage, all five models exhibit hierarchical stepped fractures. The models doped with 0.2 wt%, 0.4 wt%, 0.6 wt%, and 0.8 wt% Zr show upward and rightward shifts in their CF section curves, as illustrated in Figure 2d. When the Zr content reaches 0.6 wt%, the curve offset in the CD* section is the most pronounced compared to the other doped models, while the differences in the D*E* section and the E*F section are less significant. Following surface Zr doping, the toughness during the CF necking fracture stage is markedly enhanced, further confirming the beneficial effect of appropriate Zr doping on the material’s mechanical properties. Specifically, the upward shift of the CF segment curve indicates that during the necking fracture process, the material must endure greater stress to reach the fracture point, thereby improving its fracture toughness. At a Zr content of 0.6 wt%, the maximum offset of the CD* segment curve suggests that at this doping level, the material exhibits the strongest resistance to deformation during the initial necking fracture stage. However, despite the lack of significant differences in the D*E* and E*F segments, it is noteworthy that as the Zr content increases further, such as at 0.8 wt%, the curves in these stages begin to show a slight downward trend. This may be associated with the adverse effects introduced by excessive Zr doping, as previously mentioned. Therefore, a comprehensive analysis of performance during the CF necking and fracture stage leads to the conclusion that in material design involving surface Zr doping, 0.6 wt% is an optimal doping level. It not only significantly enhances the material’s fracture toughness but also minimizes the introduction of adverse factors.

The analysis of the mechanism by which Zr doping influences the mechanical properties of the Cu-Zr/Al_2_Cu/Al model reveals significant insights. During the elastic deformation stage, the interface matching effect elucidates the action mechanism of Zr doping: the Cu-Zr/Al_2_Cu interface maintains overall elastic continuity through coherent or semi-coherent arrangements. Although Zr atoms induce lattice distortion, their solid solubility is extremely low, preventing substantial alterations to the intrinsic elastic characteristics of the Al/Cu matrix. At a strain rate of 0.01 ps^−1^, the pinning effect of Zr on dislocations does not significantly influence dislocation slip behavior during the elastic stage. Consequently, the elastic modulus retains the dominant characteristics of the matrix.

During the plastic deformation stage, the competitive and synergistic effects of mechanisms, such as dislocation slip and twinning deformation, elucidate the action mechanism of Zr doping. Specifically, dislocation slip results from the relative sliding of atomic planes within the material, enabling continuous plastic deformation under external forces. Conversely, twinning deformation generates a mirror-symmetric twin structure through the shear of specific crystal planes, which can more effectively coordinate plastic deformation under certain conditions. Following Zr doping, the activity of these deformation mechanisms may be significantly influenced. On one hand, Zr atoms may serve as solid solution strengthening agents, impeding dislocation movement through lattice distortion, thereby enhancing the yield strength and work-hardening capacity of the material. On the other hand, the introduction of Zr may alter the stacking fault energy of the material, thus affecting the competitive relationship between dislocation slip and twinning deformation. A reduction in stacking fault energy may render twinning deformation more favorable, as it can achieve greater plastic strain with lower energy consumption [31]. In the Zr-doped materials examined in this study, an increase in Zr content may lead to subtle changes in the balance between the mechanisms of dislocation slip and twinning deformation. When the Zr content is low (ranging from 0.2 wt% to 0.6 wt%), an appropriate amount of Zr doping can enhance the synergistic effects between dislocation slip and twinning deformation, thereby enabling the material to maintain favorable mechanical properties during the plastic deformation stage. However, at higher Zr content levels (such as 0.8 wt%), an excessive concentration of Zr atoms may lead to detrimental grain boundary effects or stress concentration phenomena. These factors can impede the activity of dislocation slip and twinning deformation, ultimately resulting in a reduction in the material’s load-bearing capacity during the later stages of localized deformation.

During the necking and fracture stages, the competitive and synergistic effects of dislocation slip, twinning deformation, grain boundary sliding, and other mechanisms are particularly significant. As the Zr content increases, the difficulty of dislocation slip and twinning deformation may change, thereby affecting the fracture toughness of the material. Concurrently, the activity of grain boundary sliding may also be influenced by Zr doping, subsequently impacting the overall deformation behavior of the material. When the Zr content is at 0.6 wt%, these mechanisms may reach an optimal balance, resulting in the material exhibiting the strongest anti-deformation ability and the highest fracture toughness during the necking and fracture processes. However, with further increases in Zr content, excessive doping may disrupt this balance, leading to a decline in the material’s performance [32]. Therefore, a comprehensive understanding of the competitive and synergistic effects of these mechanisms is crucial for optimizing the mechanical properties of Zr-doped materials. By adjusting factors such as doping levels and heat treatment processes, the activity of these mechanisms can be further regulated to achieve optimal material properties.

**Figure 3:**
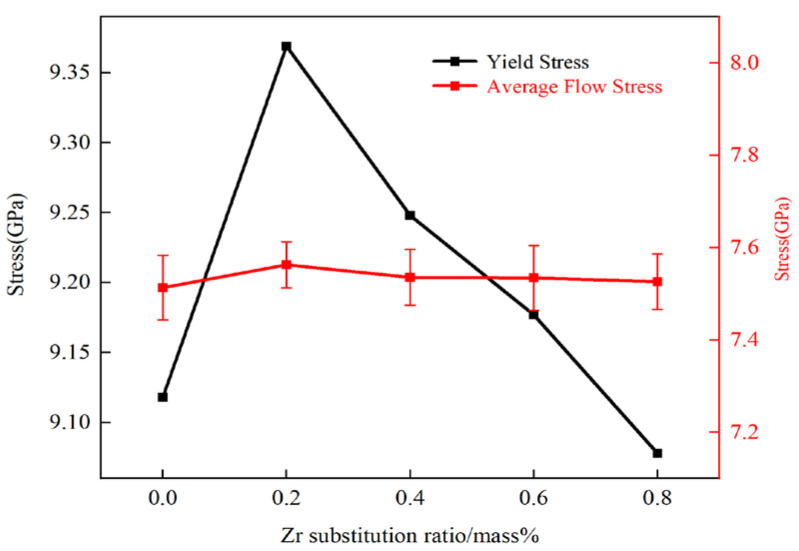
Relationship between ultimate tensile strength and flow stress with Zr content.

Figure 3 illustrates the relationship between ultimate tensile strength and flow stress with respect to Zr content. Flow stress is commonly used to describe the strength of plastic deformation, and in this study, the flow stress was obtained by fitting the average stress values within a strain range of 0.1 to 0.17 [33]. The flow stress follows the same trend as ultimate tensile strength: increasing with Zr content up to 0.2 wt% and decreasing monotonically from 0.2 to 0.8 wt%. This suggests that Zr also influences the flow stress of the model.

#### 3.1.2. Local Structural Changes During Tensile Deformation at Vertical Interfaces

The post-processing visualization software Open Visualization Tool (OVITO, Version 3.10.0), developed by Alexander Stukowski at the Institute of Materials Science, Technical University of Darmstadt in Germany, was utilized in this study to determine phase structures and defect configurations by identifying atomic stacking modes. However, it is unable to accurately identify cubic crystal systems. For instance, when the face-centered cubic (FCC) lattice is distorted, and some atoms deviate from their equilibrium positions, dislocations or defects may form, which are subsequently misclassified as Other structures by OVITO. Consequently, the phase structures identified by OVITO may actually represent dislocations or defects, thus failing to accurately reflect the true phase structure.

**Figure 4:**
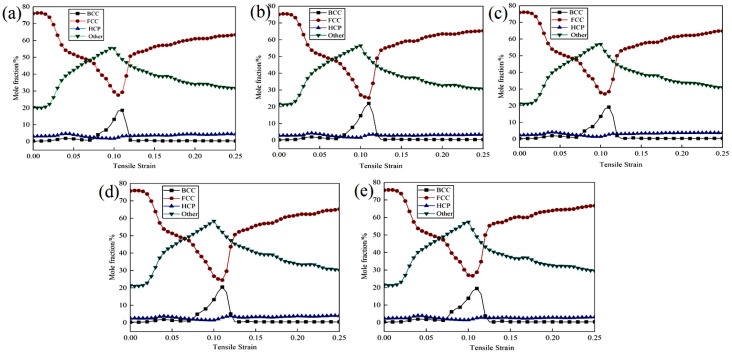
Phase structure evolution during vertical interfacial stretching of Cu-Zr/Al_2_Cu/Al doped with different Zr content: (**a**) Cu-Zr/Al_2_Cu/Al; (**b**) Cu-0.2Zr/Al_2_Cu/Al; (**c**) Cu-0.4Zr/Al_2_Cu/Al; (**d**) Cu-0.6Zr/Al_2_Cu/Al; (**e**) Cu-0.8Zr/Al_2_Cu/Al.

Plastic deformation is typically accompanied by microstructural changes, including dislocation movement, phase transformation, and twinning. Different phases may exhibit varying responses during these processes. The face-centered cubic (FCC) structure, characterized by a greater number of slip systems, is more susceptible to dislocation slip and generally demonstrates superior plastic deformation capabilities. In contrast, although the body-centered cubic (BCC) structure also possesses numerous slip systems, dislocation movement in this structure may encounter more obstacles, resulting in higher strength but lower plasticity compared to FCC. The close-packed hexagonal (HCP) structure, with fewer slip systems, tends to rely more on twinning during deformation, leading to weaker plastic deformation capabilities and a higher propensity for brittle fracture. Figure 4 illustrates the phase structure evolution of Cu-Zr/Al_2_Cu/Al vertical interfaces with varying Zr contents during the tensile process. As depicted in Figure 4a–e, the overall trends in the phase structures of the five models remain consistent throughout the tensile deformation process: the FCC phase initially decreases before increasing, the Other phase first increases and then decreases, and the BCC phase exhibits a minor peak, while the HCP phase remains nearly unchanged. During the elastic deformation stage, the FCC structure predominates, constituting 76% of the total; the BCC structure comprises approximately 0.35%; the HCP structure accounts for around 2.65%; and the Other-type (Other) structure makes up about 21%. This distribution is attributed to the initial model, where both single-crystal Cu and single-crystal Al are face-centered cubic structures (FCC), while Al_2_Cu adopts a tetragonal crystal system. At the onset of tensile deformation, the atomic arrangement within the material has not undergone significant changes, resulting in a relatively stable phase structure distribution.

As the stretching process advances into the plastic deformation stage, the FCC phase undergoes lattice distortion and local stress concentration due to deformation dominated by dislocation slip. Certain regions may experience stress-induced phase transformations, such as transitioning into the Other phase. With ongoing deformation, the stress relaxation process aids in partially restoring the FCC structure or promoting the decomposition and regeneration of the FCC phase within the Other phase, resulting in an initial decrease followed by a recovery in its proportion. The phenomenon of the Other phase initially increasing and then decreasing is attributed to high strain rates or localized high stresses that facilitate the formation of amorphous or metastable phases, including intermetallic compounds and nanocrystals. Subsequently, with continued deformation, these metastable phases become unstable and decompose into more stable phases, such as FCC. During the valley stage, where the FCC phase diminishes, local stress concentrations may trigger the formation of BCC structures such as Cu-Zr intermetallic compounds in the Zr segregation region or lead to local phase transformations and the emergence of a small peak of the BCC phase due to dislocation stacking. However, this process is relatively transient, resulting in a steep peak shape. Due to the low content and high thermodynamic stability of the HCP phase, inducing phase transformation through plastic deformation is challenging, and thus, it remains relatively stable throughout the tensile process.

**Figure 5:**
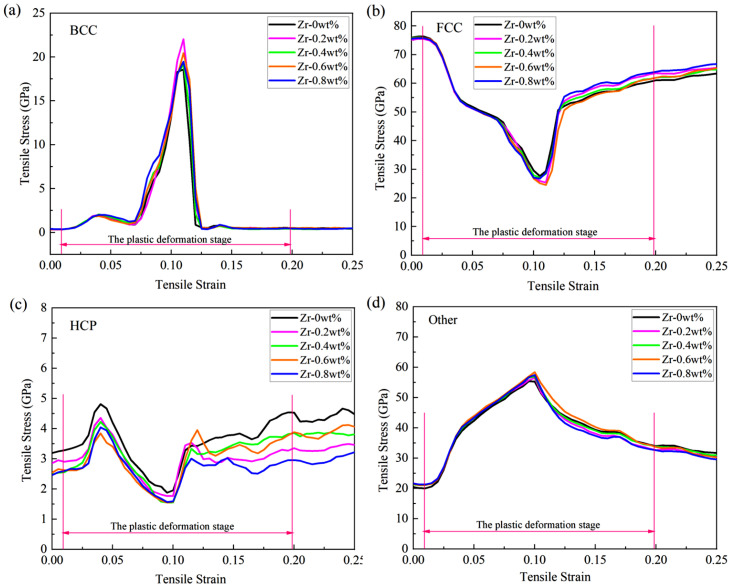
Phase structure evolution during vertical interfacial stretching of Cu-Zr/Al_2_Cu/Al doped with different Zr content: (**a**) BCC; (**b**) FCC; (**c**) HCP; (**d**) Other.

To thoroughly investigate the influence of zirconium (Zr) doping on the evolutionary trends of different phase structures in Cu-Zr/Al_2_Cu/Al composites, this study presents the evolution curves of the same phase structure during tensile deformation under various models, as illustrated in Figure 5. The findings indicate that Zr doping significantly regulates the interfacial microstructure. The curve shapes for body-centered cubic (BCC), face-centered cubic (FCC), hexagonal close-packed (HCP), and Other phases across all Zr doping concentrations (0–0.8 wt%) exhibit a high degree of consistency. The volume fraction of the body-centered cubic phase reaches its peak rapidly at a strain of ε = 0.11, with peak magnitudes following the order w_BCC(0 wt%)_ < w_BCC(0.4 wt%)_ < w_BCC(0.8 wt%)_ < w_BCC(0.6 wt%)_ < w_BCC(0.2 wt%)_. Subsequently, it declines sharply to approximately 0.5% as the strain increases to ε = 0.125. The volume fraction of the face-centered cubic phase gradually decreases from an initial value of 76% to its lowest point at ε = 0.11, with the lowest values following the order w_FCC(0.6 wt%)_ < w_FCC(0.2 wt%)_ < w_FCC(0.8 wt%)_ < w_FCC(0.4 wt%)_ < w_FCC(0 wt%)_. It then rebounds at ε = 0.125 and gradually increases with further strain. The proportion of Other phases rises from an initial 21% to a peak at ε = 0.11, with peak sizes in the order w_Other(0 wt%)_ < w_Other(0.2 wt%)_ < w_Other(0.4 wt%)_ < w_Other(0.8 wt%)_ < w_Other(0.6 wt%)_. Following this peak, the proportion gradually decreases with increasing strain. The fluctuation range of the hexagonal close-packed phase remains consistently between 1.5% and 4.8%, indicating good structural stability. The slight fluctuations observed may be attributed to the transformation of Other phases and local dislocation rearrangements.

**Figure 6:**
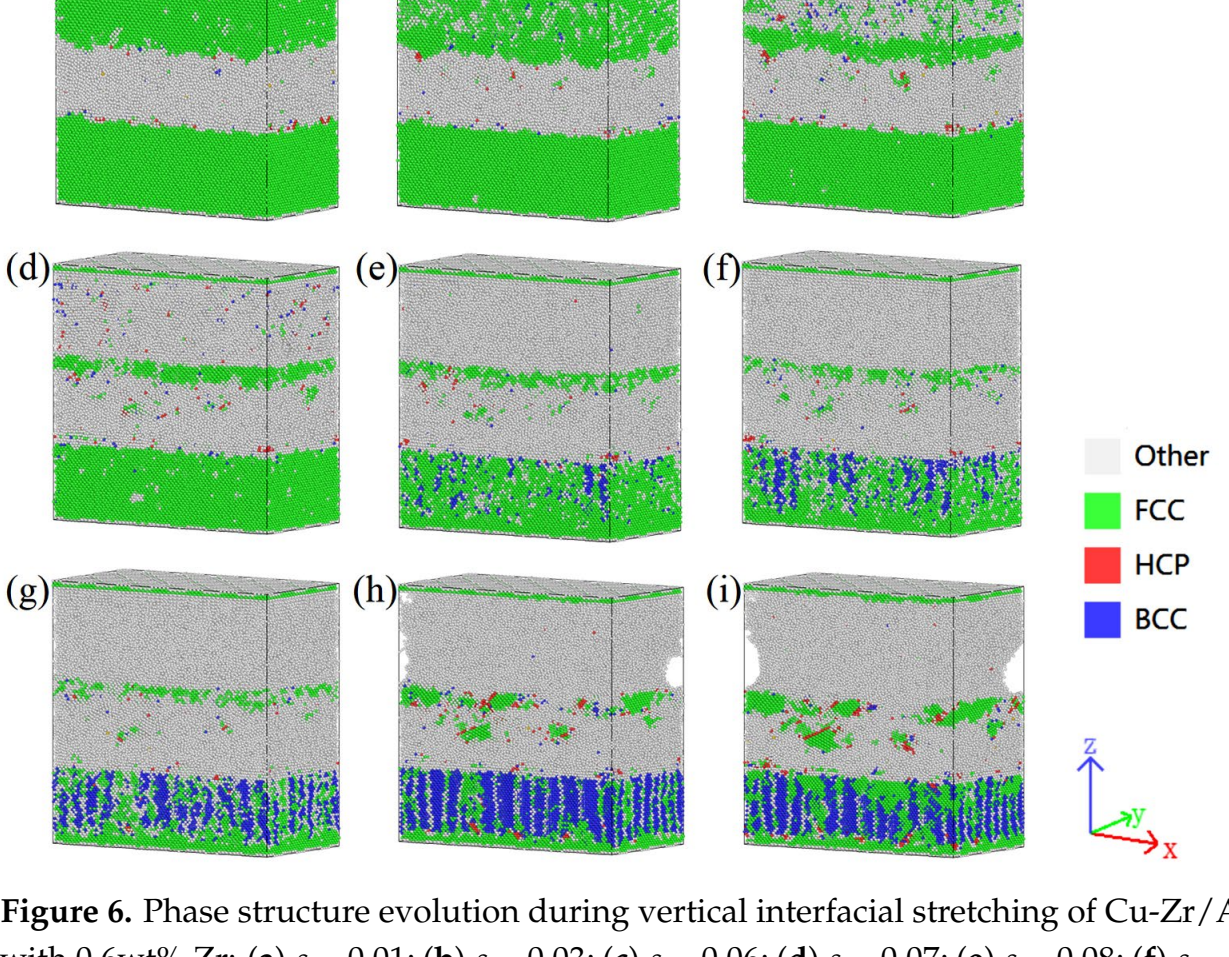
Phase structure evolution during vertical interfacial stretching of Cu-Zr/Al_2_Cu/Al doped with 0.6wt% Zr: (**a**) ε = 0.01; (**b**) ε = 0.03; (**c**) ε = 0.06; (**d**) ε = 0.07; (**e**) ε = 0.08; (**f**) ε = 0.09; (**g**) ε = 0.10; (**h**) ε = 0.11; (**i**) ε = 0.12.

The spatial distribution of various phases in the Cu-Zr/Al_2_Cu/Al model doped with 0.6wt% Zr under different strain conditions before and after tensile deformation is illustrated in Figure 6. In the initial stage of tensile deformation, the BCC and HCP phases are mainly distributed along the Al/Al_2_Cu and Al_2_Cu/Cu grain boundaries, forming fine granular or short rod-like structures, while the FCC phase, as the matrix phase, exhibits a continuous distribution. As the strain increases, compared to the Cu-Zr layer, the FCC phase in the Al layer begins to decompose first, with its continuity decreasing, mostly transforming into the Other phase and a small amount transforming into the BCC phase. After the complete decomposition of the FCC phase in the Al layer, the FCC phase in the Cu-Zr layer also begins to transform, with the majority converting into the BCC phase and a small portion into the Other phase, leading to a gradual increase in the quantities of the BCC and Other phases, which reach their peak density at ε = 0.11. Particularly in the Zr-segregated regions, the BCC and Other phases exhibit significant agglomeration, which subsequently diminishes due to dislocation annihilation and phase transformation. The Other phase primarily forms during the decomposition process of the FCC phase, and its spatial distribution is relatively random.

In regions containing Zr, the Other phase tends to aggregate at the grain boundaries of the FCC phase, forming either continuous or discontinuous network structures, which may correspond to the formation of amorphous or nanocrystalline phases. The HCP phase primarily exists as fine particles when ε < 0.11 wt%, contributing to the dispersion strengthening of the matrix, whereas short rod-like structures are observed when ε ≥ 0.11 wt%. Notably, throughout the tensile deformation process, the BCC and HCP phases maintain a close association with the Al/Al_2_Cu and Al_2_Cu/Cu grain boundaries, which may be a key factor in their formation and evolution. Furthermore, as strain continues to increase, the interfaces between the phases become more complex, and the interaction between dislocations and grain boundaries becomes more frequent, thereby promoting the formation of new phases and the transformation of existing ones. In the later stages of tensile deformation, when a significant portion of the FCC phase has transformed into the BCC phase and the Other phase, the microstructure of the entire model undergoes substantial changes, resulting in a complex multiphase structure composed of the BCC phase, the Other phase, and a small amount of the HCP phase. This structural formation not only enhances the strength and hardness of the material but also influences its toughness and plasticity.

Next, we examine the impact of different levels of Zr doping on the evolution of various phase structures. The nonlinear response of the BCC phase indicates that as the Zr content rises, the peak proportion of the BCC phase undergoes non-monotonic variations (0.2 wt% > 0.6 wt% > 0.8 wt% > 0.4 wt% > 0 wt%). This suggests that a lower Zr content (0.2 wt%) is most effective in significantly promoting the formation of the BCC phase [34]. Acting as a strong stabilizing element for the BCC phase, Zr tends to segregate at dislocations or grain boundaries at low concentrations. Through the solute drag effect, it impedes the dynamic recovery of the FCC phase. Concurrently, stress triggers the transformation of the BCC phase; for example, Cu-Zr intermetallic compounds are formed in Zr-rich areas. When the Zr content surpasses 0.2 wt%, Zr may give rise to other competing phases, or an excess of Zr could lead to lattice distortion that hinders the growth of the BCC phase, resulting in a reduction in the peak proportion. The temporary strengthening peak of the BCC phase might increase the initial work-hardening rate and delay necking; however, the subsequent sharp decline (to 0.5%) can cause local softening and may induce micro-instability.

The lowest point of the FCC phase and the competitive effect of Zr content are next examined. When ε = 0.11, the lowest proportion of the FCC phase decreases with increasing Zr content in the following order: 0 wt% > 0.4 wt% > 0.8 wt% > 0.2 wt% > 0.6 wt%. The FCC phase undergoes the most thorough decomposition at 0.6 wt% Zr. Zr doping enhances the alloy’s tendency for non-equilibrium phase transformation. Higher Zr content (0.6–0.8 wt%) promotes dislocation proliferation and local stress concentration, thereby accelerating the decomposition of the FCC phase into Other phases (such as amorphous or nanocrystalline) or into the BCC phase. However, excessive Zr (0.8 wt%) may delay dynamic recrystallization due to solute supersaturation, consequently slowing the FCC decomposition rate. Therefore, the FCC content is lowest at 0.6 wt%. The dynamic reduction-recovery behavior of the FCC phase may induce multi-stage work hardening and enhance uniform elongation. However, excessive decomposition of the FCC phase (as observed at 0.6 wt% Zr) may weaken the matrix’s plastic coordination ability, leading to a tendency for brittleness.

The dependence of the Other phase on Zr content reveals that as the Zr concentration increases, the proportion of the Other phase also rises in the order of 0.6 wt% > 0.8 wt% > 0.4 wt% > 0.2 wt% > 0 wt%. This observation suggests that Zr doping promotes the transition from single-crystal to polycrystalline structures [35,36]. Notably, the capacity to generate amorphous or stable phases is maximized at 0.6 wt% Zr. The high mixing entropy effect associated with Zr promotes shear localization and amorphization. At 0.6 wt%, the interaction between Zr segregation and the Al/Cu matrix reaches a critical threshold, resulting in the formation of a significant number of stable phases, such as Cu-Zr amorphous or nanocrystalline structures. However, when the Zr content exceeds 0.6 wt% (for instance, at 0.8 wt%), the supersaturation of solute may lead to phase separation or the precipitation of competing phases, which hinders the accumulation of the Other phase. The initial increase in the Other phase can enhance crack growth resistance due to the energy absorption properties of the amorphous phase; however, its subsequent decomposition may create a softening zone, thereby reducing fracture toughness, particularly in the sample with 0.6 wt% Zr.

The proportion of the hexagonal close-packed (HCP) phase remains consistently stable, ranging from 1.5% to 4.8%, with no significant correlation observed with zirconium (Zr) content. This stability suggests that the formation of the HCP phase is predominantly governed by thermodynamic factors rather than by plastic deformation mechanisms. The HCP phase is characterized by a high melting point and low interfacial energy, leading to its preferential precipitation during solidification. As a rigid particle, the HCP phase impedes dislocation movement during plastic deformation; however, it is challenging for the phase to undergo transformation or dissolution independently. Acting as a dispersion-strengthening phase, the HCP phase enhances the strength of the matrix. Nonetheless, its limited plastic coordination ability may serve as a nucleation site for micro-cracks, particularly at elevated Zr concentrations (>0.8 wt%), which heightens the risk of brittle fracture.

In summary, Zr doping is crucial in regulating the dynamic evolution of the phase structure in Cu-Zr/Al_2_Cu/Al composites, employing mechanisms such as solute dragging, promotion of amorphization, and competition in phase transitions. Regarding the strength–plasticity trade-off, at low Zr concentrations (0.2 wt%), the strengthening effect of the body-centered cubic (BCC) phase is predominant, exhibiting a high work-hardening rate with minimal FCC decomposition and improved plastic retention, making it suitable for applications requiring high strength and medium plasticity. At medium Zr concentrations (0.6 wt%), the presence of Other phases and significant amorphization leads to the highest peak strength; however, excessive FCC decomposition results in a marked reduction in plasticity, making it appropriate for damage-resistant applications. At high Zr concentrations (0.8 wt%), solute supersaturation inhibits the efficiency of phase transitions, yielding performance metrics that lie between those observed at the previous two concentrations. This increase in the HCP phase may further compromise toughness, and the associated performance gains are limited, warranting cautious application. Future research could explore gradient Zr doping or multi-scale second-phase design to better coordinate the contributions of each phase evolution to the mechanical properties.

**Figure 7:**
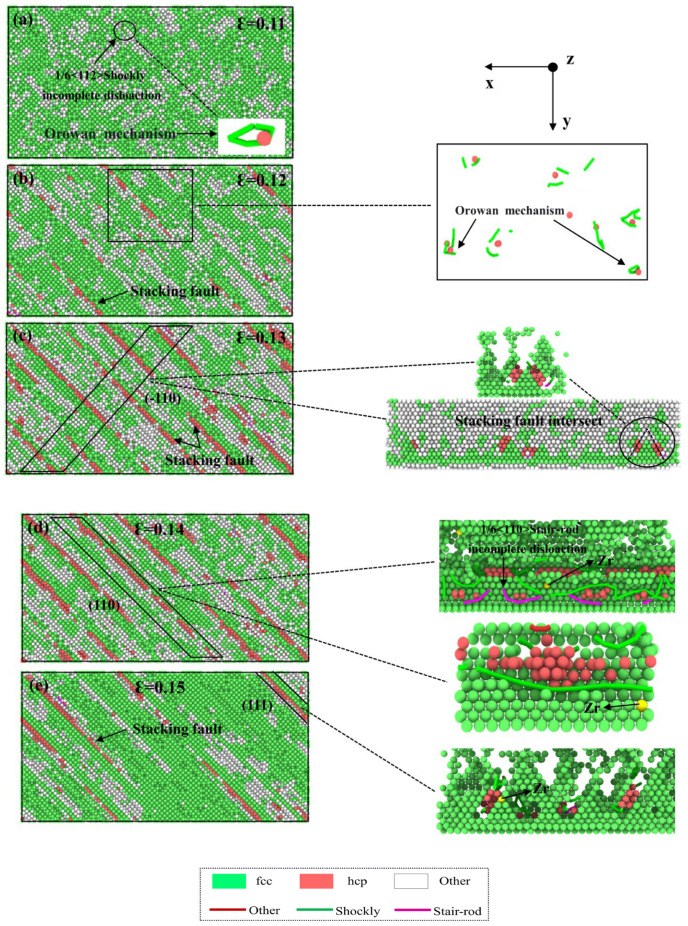
Atomic diagrams of Cu-Zr/Al_2_Cu/Al doped with 0.2wt% Zr at different strains during vertical interfacial tensile process: (**a**) ε = 0.11; (**b**) ε = 0.12; (**c**) ε = 0.13; (**d**) ε = 0.14; (**e**) ε = 0.15.

Figure 7 illustrates the DXA analysis of the Cu layer parallel to the interface direction in the Cu-0.2Zr/Al_2_Cu/Al model during the plastic deformation stage. The combination of stress–strain curves and DXA analysis provides insights into the structural changes and strengthening mechanisms in the Cu layer. Figure 7a illustrates the dispersion of “Other” structures under the influence of the stretching load during the plastic deformation stage at ε = 0.11. This dispersion arises from the disorderly arrangement of atoms in the Cu matrix at normal lattice positions due to tension, leading to a notable increase in vacancies, dislocations, and Other defects, accompanied by an augmentation in the “Other” atom content. Additionally, localized regions exhibit 1/6<112> Shockley partial dislocations that traverse atomic vacancy loops. Conversely, this phenomenon is absent in the Zr-undoped Cu/Al_2_Cu/Al model during stretching, indicating the presence of a second phase dispersed within the matrix phase. The interaction between this second phase and dislocations fortifies the model’s resistance to deformation by impeding dislocation motion, constituting the Orowan strengthening mechanism. Therefore, Zr doping in the Cu matrix facilitates Orowan strengthening. At ε = 0.12, as depicted in Figure 7b, the Other structures gradually aggregate along the <111> crystal direction, accompanied by a minor generation of HCP structure. The Other structure content increases with strain, while the appearance of HCP structure suggests the emergence of stacking faults along the <111> crystal direction. Simultaneously, the slip system of the Cu layer occurs on the (111) crystal plane, heightening the Orowan strengthening effect under these conditions. At ε = 0.13, Figure 7c demonstrates a sharp rise in stacking fault content within the Cu matrix. Stacking faults are observed to be parallel or intersecting on the (−110) surface, accompanied by the presence of two types of dislocations near the stacking faults: 1/6<112> Shockley partial dislocations and 1/6<110> Stair-rod partial dislocations, indicative of cross-slip characteristics. Compared to Figure 7a,b, the Other structures transition from a relatively dispersed form to clustered clusters, distributed between stacking faults or at the intersection of stacking faults. At ε = 0.14, the stacking fault content reaches its peak, and stair-rod partial dislocations are observed on the (111) surface, resulting from dislocation reactions at the stacking fault [37]:

(1) $$\frac{1}{6}\left[2\overset{-}{1}\overset{-}{1}\right]+\frac{1}{6}\left[\overset{-}{1}\overset{-}{1}0\right]\to \frac{1}{6}\left[1\overset{-}{2}\overset{-}{1}\right]$$

The distribution of Zr near the stacking faults and dislocation lines can be observed from Figure 7d,e. This observation indicates the influence of Zr distribution on the occurrence of stacking faults and the initiation of dislocations, particularly at the intersection of stacking faults. Compared to the model without Zr doping, the presence of Zr facilitates the generation of stacking faults and the initiation of dislocations. At ε = 0.15, the HCP and Other structures decline significantly in the Cu matrix. This is attributed to the lower tensile strength of Al compared to Cu. As the stretching process progresses, the Al layer starts to fracture. Consequently, the stress imposed on the Cu layer gradually diminishes during the fracture of the Al layer, leading to the progressive disappearance of stacking faults, vacancies, and other defects.

#### 3.1.3. Analysis of Dislocation Density During Tensile Deformation at Vertical Interfaces

**Figure 8:**
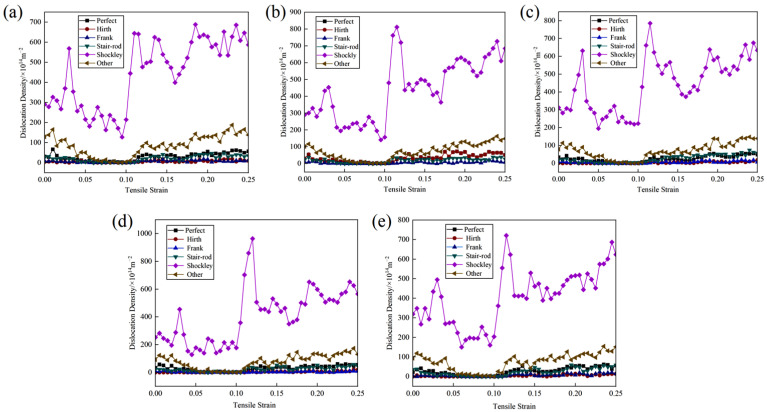
Variation of dislocation length density with strain during vertical interfacial tension in Cu-Zr/Al_2_Cu/Al model doped with different Zr content: (**a**) Cu-Zr/Al_2_Cu/Al; (**b**) Cu-0.2Zr/Al_2_Cu/Al; (**c**) Cu-0.4Zr/Al_2_Cu/Al; (**d**) Cu-0.6Zr/Al_2_Cu/Al; (**e**) Cu-0.8Zr/Al_2_Cu/Al.

The strain-dependent evolution of dislocation length density in the Cu-Zr/Al_2_Cu/Al model, doped with various Zr concentrations across the interface, is depicted in Figure 8. Notably, Shockley partial dislocations emerge as the predominant dislocation type during the tensile deformation of the model. Initially, in the initial stage of plastic deformation, the density of Shockley partial dislocations exhibits fluctuations and escalates with increasing strain, reaching a pronounced peak at 0.03 strain. Afterward, it decreases with fluctuations and then stabilizes between the yield points, indicating substantial dislocation consumption.

In the intermediate stage of plastic deformation, the dislocation density of Shockley partial dislocations rises rapidly, reaching a maximum near the yield point. The maximum dislocation density varies with different Zr doping levels, attaining its peak value of 963.42 × 1014 m^−2^ at a Zr doping content of 0.6 wt%. Thereafter, the dislocation density curve stabilizes with fluctuating decline, reflecting the combined influence of dislocation nucleation and glide. Remarkably, dislocation starvation predominates the plastic deformation process.

### 3.2. Tensile Deformation at Parallel Cu-Zr/Al_2_Cu/Al Interfaces

#### 3.2.1. Influence of Different Zr Content on Tensile Deformation at Parallel Cu-Zr/Al_2_Cu/Al Interfaces

**Figure 9:**
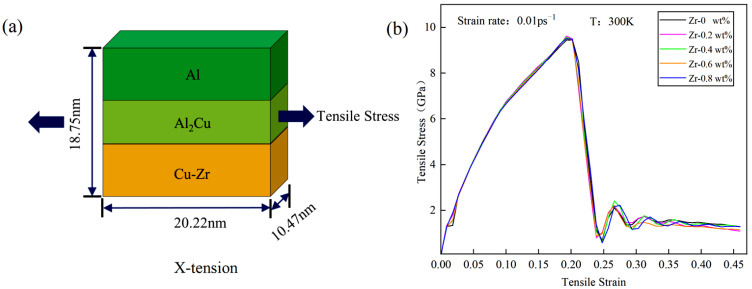
Tensile model and stress–strain curve of Cu-Zr/Al_2_Cu/Al parallel interface with different Zr content: (**a**) tensile deformation model of Cu-Zr/Al_2_Cu/Al parallel interface; (**b**) stress–strain curves of Cu-Zr/Al_2_Cu/Al parallel interface under tensile deformation.

This section explores the tensile deformation behavior of five Cu-Zr/Al_2_Cu/Al models with varying Zr doping levels at room temperature. Figure 9a provides a schematic of the tensile model, illustrating uniaxial stretching along the x-axis at a strain rate of 0.01 ps^−1^ and a target strain of 50%. Interatomic interactions within the model are characterized using the embedded atom method (EAM) [38].

Figure 9b illustrates the stress–strain curves of Cu-Zr/Al_2_Cu/Al with varying zirconium contents under x-axis stretching. Notable similarities are evident during the tensile deformation process across the five models. In the strain range of 0 to 0.01, the stress–strain relationship exhibits linear growth, indicative of elastic deformation. As the strain increases from 0.01 to 0.2, the material transitions into the plastic deformation stage. At a strain of 0.19, the stress for all five materials reaches a peak of approximately 9.5 GPa, exhibiting characteristics of ductile fracture following the attainment of maximum tensile strength. With further strain increase, the stress rapidly declines to a range of 0.6 to 0.8 GPa, after which a second peak emerges on the stress–strain curve. Ultimately, as the strain continues to increase, the stress demonstrates a fluctuating decrease.

#### 3.2.2. Local Structural Changes During Tensile Deformation at Parallel Interfaces

**Figure 10:**
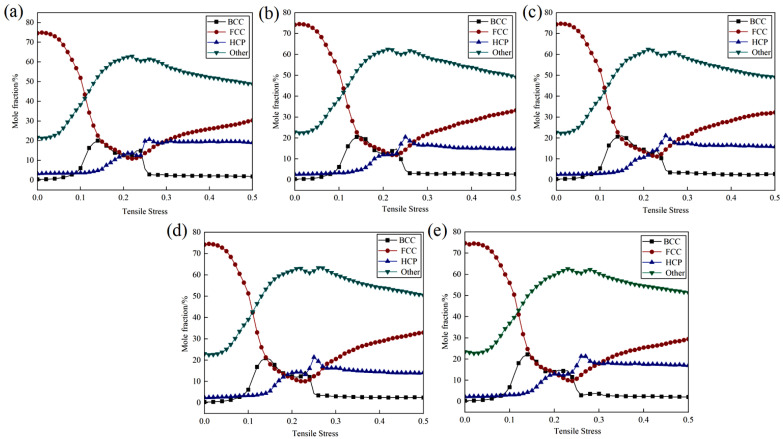
Phase structure evolution during the tensile process of Cu-Zr/Al_2_Cu/Al parallel interface doped with different Zr content: (**a**) Cu-Zr/Al_2_Cu/Al; (**b**) Cu-0.2Zr/Al_2_Cu/Al; (**c**) Cu-0.4Zr/Al_2_Cu/Al; (**d**) Cu-0.6Zr/Al_2_Cu/Al; (**e**) Cu-0.8Zr/Al_2_Cu/Al.

Figure 10 depicts the evolution of phase structure throughout the tensile testing of Cu-Zr/Al_2_Cu/Al parallel interfaces with varying Zr contents. By analyzing the stress–strain curves, it becomes evident that during the plastic deformation stage, particularly when the strain is between 0.01 and 0.1, the proportion of FCC structures gradually decreases. Conversely, the proportions of BCC and HCP structures show a gradual increase, while the content of Other structures significantly rises. This occurrence can be ascribed to the tensile process, where certain Cu atoms within the FCC structure undergo lattice distortion, resulting in their conversion into BCC and HCP structures. This transformation is accompanied by the emergence of a considerable amount of disordered structures, classified as Other structures. These microstructural alterations are key contributors to the observed plastic deformation in the materials.

As the dependent variable continues to increase, particularly when it ranges from 0.1 to 0.19, the material progresses to a more advanced stage of plastic deformation. At this point, the proportion of the FCC structure significantly decreases, whereas the proportions of BCC and Other structures notably increase. Furthermore, the HCP structure exhibits a more pronounced upward trend compared to the elastic stage. These observations suggest that in the later stages of plastic deformation, the material’s microstructure undergoes more substantial changes, with the FCC structure transforming further into BCC, HCP, and Other structures. The increase in the BCC and HCP structures may indicate the formation of a greater number of dislocations and stacking faults within the material. These defect structures are crucial for enhancing the material’s plastic deformation capacity. Additionally, the continuous rise in the proportion of Other structures may reflect an increasing degree of disorder within the material during the stretching process. This disorder is advantageous, as it allows the material to absorb more energy during the plastic deformation phase, thereby enhancing its toughness.

Further analysis of the phase structure evolution diagram reveals that the incorporation of Zr into the Cu layer facilitates the transformation from FCC to BCC during the plastic deformation stage. As the Zr doping concentration increases, the BCC content correspondingly rises. The trends in phase structure evolution for Cu-Zr/Al_2_Cu/Al parallel interfaces with varying Zr contents exhibit similarities during the tensile process; however, the specific changes in content differ. At a Zr doping level of 0.8 wt%, the BCC content reaches its maximum value of 22.19% at a strain of 0.14. This finding indicates the existence of an optimal Zr doping concentration that allows the material to achieve the highest BCC structure formation during plastic deformation, potentially resulting in superior mechanical properties. This further substantiates the significant influence of Zr doping on the evolution of the phase structure within the Cu layer during the parallel stretching process, highlighting Zr’s critical role as an effective alloying element in regulating material microstructure and enhancing material properties.

#### 3.2.3. Distribution of Atomic Stress and Dislocation Lines During Tensile Deformation at Parallel Interfaces

**Figure 11:**
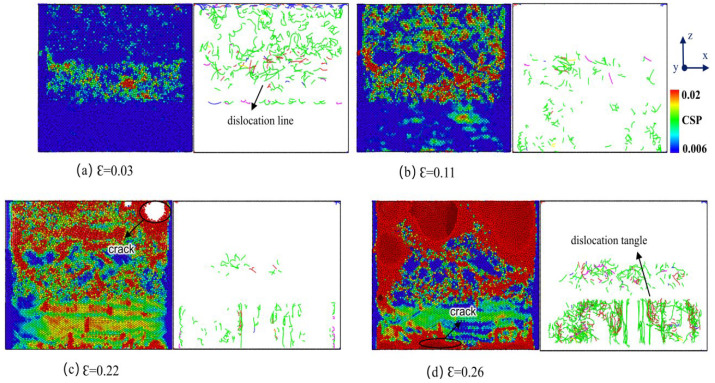
Atomic structure evolution and dislocation line distribution during Cu-Zr/Al_2_Cu/Al tensile process doped with 0.2 wt% Zr.

Figure 11 depicts the distribution of atomic stress and dislocation lines in the Cu-Zr/Al_2_Cu/Al alloy, which contains 0.2 wt% Zr, throughout the tensile process. By synthesizing these data with the stress–strain curve, a thorough analysis of the tensile behavior can be conducted. In the early stages of plastic deformation (ε = 0.03), the atomic stress within the Al_2_Cu layer significantly increases, suggesting that the tensile load is conveyed to this layer via the Al/Al_2_Cu interface, initiating local brittle deformation. The Al layer undergoes dislocation slip preferentially because of its lower yield strength, whereas the Al_2_Cu layer builds up elastic strain energy due to stress concentration at the interface. At ε = 0.11, the dislocation lines within the Al layer disappear. At the Al/Al_2_Cu interface, micro-cracks begin to form due to the brittleness of the Al_2_Cu phase; however, interfacial dislocations continue to partially mitigate the deformation. As ε reaches 0.22, the Al layer fractures from necking, and the Al/Al_2_Cu interface completely fails as micro-cracks propagate. The load is then shifted to the Cu layer and the remaining Al_2_Cu layer. When ε surpasses 0.24, the Zr-doped Cu layer triggers a dynamic Hall–Petch effect via dislocation entanglement, leading to a secondary hardening peak on the stress–strain curve. In the end, the Cu layer succumbs to failure due to interface debonding and dislocation channeling.

Figure 11 clearly illustrates that the introduction of Zr element substantially influences the dislocation line distribution within the Cu-Zr/Al_2_Cu/Al alloy as strain increases. Notably, within the Cu layer, the addition of Zr results in more intricate dislocation line movement and entanglement. Acting as a solid solution strengthening agent, Zr atoms bolster the strength and toughness of the Cu layer by obstructing dislocation line movement. Additionally, the inclusion of Zr alters the characteristics of the Cu/Al_2_Cu interface, leading to a more uniform dislocation line distribution at the interface, which in turn postpones the failure process of the interface. These findings offer essential understanding of the impact of Zr on the tensile deformation behavior of the Cu-Zr/Al_2_Cu/Al alloy.

### 3.3. Effect of Strain Rate on Tensile Deformation of Cu-0.2Zr/Al_2_Cu/Al Interfaces

The effect of various strain rates on the tensile deformation of Cu-0.2Zr/Al_2_Cu/Al was investigated, with four different strain rates (0.0005 ps^−1^, 0.001 ps^−1^, 0.005 ps^−1^, and 0.01 ps^−1^) employed as detailed in Table 3. Uniaxial stretching was performed along the z-axis with a target strain of 30%, and the interactions between individual atoms in the model were modeled using the embedded atom method (EAM).

**Figure 12:**
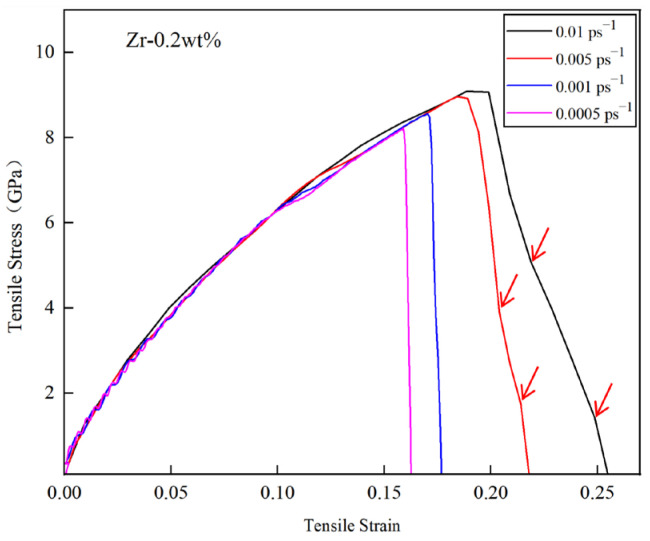
The stress–strain curves of Cu-0.2Zr/Al_2_Cu/Al interface model for difference strain rates at 300 K.

Figure 12 illustrates the tensile stress–strain curves of the Cu-0.2Zr/Al_2_Cu/Al interface model at various strain rates at 300 K. It is evident from the figure that models subjected to different strain rates exhibit brittle fracture characteristics.

During the elastic deformation stage, the mechanical properties exhibit minimal variation across different strain rates. However, as the strain rate increases, the adjacent base metals become tightly coupled, enhancing the coordination of deformation and significantly increasing both the ultimate tensile strength and plasticity. This phenomenon occurs during the tensile deformation process, wherein the A1 layer, characterized by a low yield strength, reaches its yield point first and undergoes plastic deformation, while the adjacent Cu-0.2Zr layer remains in the elastic deformation stage. As the load continues to increase, and the yield strength of the Cu-0.2Zr/Al_2_Cu/Al interface model is attained, the Cu-0.2Zr layer begins to undergo plastic deformation. Given that the elongation of the Cu-0.2Zr layer is less than that of the A1 layer, a layered structure is established. The intermediate interface layer facilitates the transfer of stress generated by the Cu-0.2Zr layer to the A1 layer, alleviating stress concentration within the Cu-0.2Zr layer and enhancing the overall elongation and tensile strength of the material.

It is important to note that the simulation software employed has certain limitations regarding time settings, resulting in a strain rate used in this calculation that is significantly higher than that utilized in actual tensile experiments. This elevated strain rate can considerably influence the mechanical properties of the material, including enhanced yield strength and varying fracture modes. In comparison to the stress–strain curve typically observed in the cast-rolled state during experiments, the results derived from this study reveal novel alterations.

When the simulated strain rate is between 0.0005 ps^−1^ and 0.01 ps^−1^, the stress–strain curve of the Cu-0.2Zr/Al_2_Cu/Al layered heterostructure exhibits several fluctuations during the initial stage of strain increase in the elastic deformation region. As the tensile strain continues to increase, the Cu-0.2Zr/Al_2_Cu/Al layered heterostructure transitions into the plastic deformation region. Following the attainment of the ultimate tensile strength, the stress rapidly declines to zero, resulting in overall fracture. Conversely, when the strain rate ranges from 0.005 ps^−1^ to 0.01 ps^−1^, the stress–strain curve in the initial stage of strain increase does not exhibit fluctuations. As the tensile strain continues to rise, the Cu-0.2Zr/Al_2_Cu/Al layered heterostructure also enters the plastic deformation region. After reaching the ultimate tensile strength, the stress decreases gradually in a step-like manner, as indicated by the red arrow in Figure 12. This behavior suggests that interface separation and delamination fracture occur in the composite material during the stretching process.

**Figure 13:**
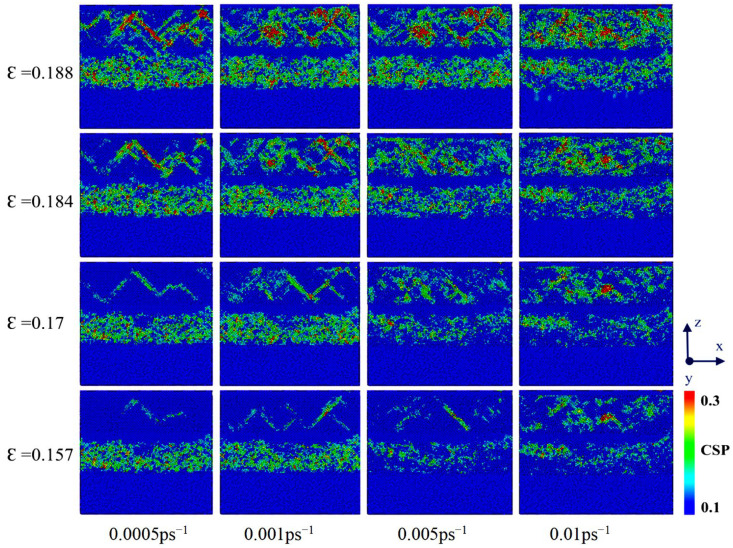
The atomic structure of the interface configuration under compressive load for the strain rate of 0.001 ps^−1^.

Figure 13 illustrates the atomic stress diagram during the stretching process at various strain rates. The results indicate that at low strain rates of 0.0005 ps^−1^ to 0.01 ps^−1^, dislocations initially begin within the intermetallic compound (IMC) Al_2_Cu layer as the model gradually approaches its ultimate tensile strength under tensile load. In the early stages of stretching, the dislocation motion is relatively slow, allowing atoms sufficient time to move and rearrange in response to external strains. As the strain increases, the dislocation density in the Al layer progressively increases, leading to the formation of dislocation tangles that impede dislocation movement, resulting in local stress concentrations and serving as a source for crack initiation. When the applied tensile stress surpasses the local strength of the material, microcracks begin to form and propagate. Given that the fracture strength of copper exceeds that of aluminum, cracks preferentially occur within the aluminum matrix. Furthermore, aluminum exhibits a typical face-centered cubic crystal structure, which possesses numerous slip systems, thereby providing good plasticity and a propensity for substantial plastic deformations. In contrast, the Al_2_Cu layer is relatively brittle, making it susceptible to grain boundary or transgranular fractures. Additionally, there is a coupling effect at the phase interface between the Al_2_Cu layer and the copper–aluminum matrix. This structural characteristic enhances the bonding strength and deformation coordination of the Cu-0.2Zr/Al_2_Cu/Al composite. Consequently, the stress–strain curve exhibits multiple fluctuations during the initial stages of tension. After reaching the ultimate tensile strength, the material fails as a whole, resulting in a rapid drop in stress to zero. This behavior aligns with the observed trends in the stress–strain curve during the vertical interface tensile deformation process of the Cu/Al_2_Cu/Al layered heterostructure [29].

At high strain rates of 0.005 ps^−1^ to 0.01 ps^−1^, atoms are unable to adapt to external strains rapidly enough, which limits the movement rate of dislocations. This limitation results in dislocation accumulation and aggregation, thereby increasing the occurrence of stress concentrations and exacerbating dislocation formation and movement. Additionally, the slip path of dislocations is hindered, making dislocation movement more challenging. As the density of dislocations continues to increase, high-density dislocation lines emerge in a strain rate hardening state, which enhances the ultimate tensile strength of the material. As the strain rate further increases, significant stress concentrations develop in the interface area, leading to the initial separation of Cu-0.2Zr/Al_2_Cu/Al along the layer interface, followed by sequential fracture of each layer. In room-temperature tensile property experiments on the Cu/8011/1060 multi-metal composite prepared with a rolling reduction rate of 38%, delamination fracture was observed. In this case, the upper surface layer of copper, the middle layer of 8011 aluminum alloy, and the interface between the 1060 aluminum alloy in the lower surface layer first separated, followed by sequential fracture of each matrix layer. Combining the actual in situ tensile results with observations of the room-temperature tensile fracture cross-sectional structure revealed that after local delamination occurs in the interface area, the bond between Al_2_Cu and the matrix is disrupted, partially losing the constraint effect of the interface area. This results in the generation of stress concentrations between the Cu layer and the Al layer. As strain increases, the crack front detaches from the Cu layer and propagates into the Cu layer until it ultimately fractures. Following the fracture of the Cu layer, the crack extends into the Al layer, resulting in the complete failure of the composite plate.

In practical engineering applications, it is essential to design materials that can adapt to specific loading conditions. High-strength and high-conductivity copper–aluminum layered composite materials have found applications in electrical engineering, transportation, the microelectronics industry, and aviation owing to their exceptional performance. The aerospace sector and other fields present broad application prospects for these materials. This research elucidates the regulatory mechanism of zirconium (Zr) doping on the evolution of phase structure and the mechanical properties of Cu-Zr/Al_2_Cu/Al composites. The findings provide direct guidance for the design and optimization of high-performance composites in aerospace applications. By varying the amount of Zr doping (0.2 wt% Zr), the dislocation storage capacity at the Al/Al_2_Cu interface is optimized, leading to a delay in interface failure and an enhancement in the fatigue life of the multilayer composite. Furthermore, the dynamic work-hardening effect induced by the FCC-to-BCC phase transformation (evidenced by a BCC peak at ε = 0.11) significantly improves the energy absorption capacity of the material under impact loads, making it suitable for components subjected to bird strikes or vibrational loads. Additionally, composite materials containing 0.6 wt% Zr can also be engineered. By pinning grain boundaries with high-density secondary phases (amorphous/nanocrystalline), the high-temperature creep resistance can be enhanced, which is particularly beneficial for components such as engine nacelles. In addition to Zr, the incorporation of trace amounts of chromium (Cr) into the copper matrix as well as trace amounts of iron (Fe), silicon (Si), and other alloying elements into the aluminum matrix can further enhance their performance.

The current study concentrates on room-temperature conditions to clarify the fundamental phase stability and strengthening mechanisms within the Cu-Zr/Al_2_Cu/Al system. Our simulations revealed that the FCC-to-BCC transition is suppressed under strain at 300 K. However, temperature-dependent effects, such as accelerated dynamic recovery and Zr diffusion at higher temperatures, or strain-localized BCC formation coupled with Al_2_Cu interfacial embrittlement at cryogenic conditions, still need to be quantified. The thermal expansion mismatch between Al and Cu-Zr layers introduces residual stresses that could critically affect phase selection, particularly through compressive stress-induced FCC stabilization at high temperatures versus tensile stress-driven BCC nucleation during cooling. Additionally, temperature-modulated Zr diffusion could simultaneously weaken solid solution strengthening and enhance amorphous phase retention at specific compositions. Future temperature-dependent molecular dynamics simulations will quantify the thermal effects on phase equilibria and interfacial stresses.

## 4. Conclusions

This study employed molecular dynamics (MD) simulations to investigate the deformation behavior of Cu-Zr/Al_2_Cu/Al models with varying Zr content. The simulations covered vertical and parallel interface tensile deformations as well as vertical interface deformation under different strain rates. The influence of Zr content and strain rate on ultimate tensile strength was examined along with the atomic structural evolution and dislocation dynamics during deformation. The Cu-Zr/Al_2_Cu/Al model exhibited a brittle fracture mechanism under vertical interface tensile deformation. Incorporating a small amount of Zr (0–0.8 wt%) moderately enhanced the model’s strength and plasticity, but excessive Zr reduced its plasticity and ductility. The introduction of Zr increased the extent of local FCC-to-BCC transformation compared to the undoped model. At 0.2 wt% Zr, the model achieved a peak ultimate tensile strength of 9.369 GPa, with the highest degree of FCC-to-BCC transformation, where the BCC structure constituted 22.04% of the model at a yield strain of 0.11. Under horizontal interface tensile deformation, the Cu-Zr/Al_2_Cu/Al model demonstrated a ductile fracture mechanism. Initially, the tensile load primarily impacted the Al_2_Cu layer, and as strain increased, the load-bearing capacity extended to the Al layer, the Al_2_Cu layer, and the Al/Al_2_Cu interface. Gradually, the Al layer and the Al/Al_2_Cu interface began to fail, leading to load transfer to the Cu layer, the Al_2_Cu layer, and the Cu/Al_2_Cu interface. Eventually, with further strain accumulation, the Cu layer, the Cu/Al_2_Cu interface, and the Al_2_Cu layer underwent consecutive failures until final fracture. During vertical interface tensile deformation, strain rate significantly affected the mechanical properties of the model. Within the range of strain rates studied, the ultimate tensile strength and plasticity of the model increased with rising strain rate. The effect of strain rate on enhancing the model’s performance was highly pronounced.

## Figures and Tables

**Table 1:** Parameter setting of interface model of Cu/Al_2_Cu/Al.

Materials	Coordinate Axis	Crystal Orientation	Dimension (Å)	Cycle
Cu	X	[100]	202.1	56
	Y	[010]	104.7	29
	Z	[001]	64.4	18
Al_2_Cu	X	[100]	200.2	33
	Y	[010]	103.1	17
	Z	[001]	63.4	13
Al	X	[100]	202.5	50
	Y	[010]	105.3	26
	Z	[001]	60.7	15

**Table 2:** Mechanical properties of Cu-Zr/Al_2_Cu/Al model with different Zr content.

Zr Content/wt%	Ultimate Tensile Strength/GPa	Corresponding Strain	Modulus of Elasticity/GPa	R^2^ Value
0	9.12	0.1884	33.01	0.997
0.2	9.37	0.1982	33.53	0.995
0.4	9.25	0.1971	32.74	0.995
0.6	9.18	0.1963	32.52	0.996
0.8	9.07	0.1958	32.37	0.995

**Table 3:** Parameter setting under strain rates.

Strain Rate	Relaxation Time	Step Size	Temperature	Time Step
0.0005 ps^−1^	10 ps	0.001 ps	300 K	40,000
0.001 ps^−1^	10 ps	0.001 ps	300 K	80,000
0.005 ps^−1^	10 ps	0.001 ps	300 K	400,000
0.01 ps^−1^	10 ps	0.001 ps	300 K	800,000

## Data Availability

The original contributions presented in this study are included in the article. Further inquiries can be directed to the corresponding author.

## References

[1] Akgül B., Erden F., Özbay S. (2021). Porous Cu/Al composites for cost-effective thermal management. Powder Technol..

[2] Wu S., Cai X., Zhou L., Yang C., Li W., Cheng Y. (2022). Contribution of Mechanical Activation on the Growth of Intermetallic Compound Layers at the Cu/Al Interface During Vacuum Hot Pressing. Trans. Indian Inst. Met..

[3] Dashti A., Keller C., Vieille B., Guillet A., Bouvet C. (2021). Experimental and Finite Element Analysis of the Tensile Behavior of Architectured Cu-Al Composite Wires. Materials.

[4] Amiri F.S., Hosseinipour S.J., Aval H.J., Jamaati R. (2023). Fabrication of a novel high-strength and high-conductivity copper-clad aluminum composite wire. CIRP J. Manuf. Sci. Technol..

[5] Feng J. (2019). Texture development in the interfacial zone of Al/Cu bimetal cold roll-bonded for E-mobility. Mater. Lett..

[6] Li H., Yang Y., Liang X., Zhang W., Cao L., Wu C., Zeng Z., Wang L. (2020). Effect of annealing temperature and time on the microstructure, mechanical properties and conductivity of cold-rolled explosive Cu/Al composite sheets. Mater. Res. Express.

[7] Ebrahimi M., Liu S., Wang Q., Jiang H., Ding W., Shang Z., Luo L. (2020). Evaluation of interface structure and high-temperature tensile behavior in Cu/Al8011/Al5052 trilayered composite. Mater. Sci. Eng. A.

[8] Ebrahimi M., Liu G., Li C., Wang Q., Jiang H., Ding W., Su F. (2020). Experimental and numerical analysis of Cu/Al8011/Al1060 trilayered composite: A comprehensive study. J. Mater. Res. Technol..

[9] Ebrahimi M., Li G., Li C., Wang Q., Jiang H., Ding W., Su F., Shang Z. (2021). Characteristic investigation of trilayered Cu/Al8011/Al1060 composite: Interface morphology, microstructure, and in-situ tensile deformation. Prog. Nat. Sci. Mater. Int..

[10] Li J., Gao H., Kong C., Yu H. (2022). Insight into the bonding mechanism in Cu/Al/Cu clad sheets via introduction of thin SUS304 interlayer. J. Mater. Res. Technol..

[11] Mao Z., Xie J., Wang A., Wang W., Ma D., Liu P. (2020). Effects of annealing temperature on the interfacial microstructure and bonding strength of Cu/Al clad sheets produced by twin-roll casting and rolling. J. Mater. Process. Technol..

[12] Song H., Wei H., Mu X., Han T., Che C., Geng G. (2020). Effect of Pulse Current-Assisted Rolling on the Interface Bonding Strength and Microstructure of Cu/Al Laminated Composite. Metals.

[13] Hu Y., Chen Y., Li L., Hu H.-D., Zhu Z. (2016). Microstructure and properties of Al/Cu bimetal in liquid–solid compound casting process. Trans. Nonferrous Met. Soc. China.

[14] Chen Y., Wang A., Liu S., Xie J. (2019). Coupling effects of matrix and interface on synergetic deformation behaviors of Cu/Al composite plates. Mater. Res. Express.

[15] Wang Y., Lin X., Zhao Y., Wang Z., Yu X., Gao X., Huang W. (2023). Laser powder bed fusion of Zr-modified Al-Cu-Mg alloy: Processability and elevated-temperature mechanical properties. J. Mater. Sci. Technol..

[16] Wang Y., Cao L., Wu X., Lin X., Yao T., Peng L. (2023). Multi-alloying effect of Ti, Mn, Cr, Zr, Er on the cast Al-Zn-Mg-Cu alloys. Mater. Charact..

[17] Nam N.D., Bui T.N.M., Vu A.T., Sai M.T., Pham M.K., Tran D.H. (2022). Properties of Al-Zn-Mg-Cu alloy when modified by La, Ce, and thermal-mechanical. Acta Metall. Slovaca.

[18] Hua-xin L.I., FENG Y.-D., Shen W., Lu C., Zheng W., Ma Y., Ma G., Jin Z.-P., He Y., Yang J.-G. (2022). Microstructure and mechanical properties of Cu/Al joints brazed using (Cu, Ni, Zr, Er)-modified Al—Si filler alloys. Trans. Nonferrous Met. Soc. China.

[19] Zhang M., Wang J., Wang Q., Xue C., Liu X. (2022). Quantifying the effects of Sc and Ag on the microstructure and mechanical properties of Al–Cu alloys. Mater. Sci. Eng. A.

[20] Hu K., Zou C., Wang H., Wei Z. (2023). Influence of Ti elements on the evolution of microstructure, mechanical properties and thermal stability of Al-Cu alloy. J. Alloys Compd..

[21] Jain S., Naveen L., Kumar V., Samal S. (2023). Effect of Ni and Si alloying elements on the phase evolution, mechanical properties, tribological behaviour of Al–Cu alloys. Mater. Chem. Phys..

[22] Maмзypинa O.И., Aмep C.M., Лoгинoвa И.C., Глaвaтских M.B., Mochugovskiy A.G., Barkov R.Y., Пoзднякoв A.B. (2022). Effect of Zr on Microstructure and Mechanical Properties of the Al–Cu–Yb and Al–Cu–Gd Alloys. Metals.

[23] Deng P., Mo W., Ouyang Z., Tang C., Luo B., Bai Z. (2023). Mechanical properties and corrosion behaviors of (Sc, Zr) modified Al-Cu-Mg alloy. Mater. Charact..

[24] Park J.-W., Ahn M., Yu G., Kim J., Kim S., Shin C. (2024). Influence of alloying elements and composition on microstructure and mechanical properties of Cu-Si, Cu-Ag, Cu-Ti, and Cu-Zr alloys. Mater. Today Commun..

[25] Cai P., Zhang H., Wang K.-F., Zhang G., Chou K.-C. (2024). Effect of Ti or Zr alloying on the microstructure evolution and mechanical properties of W-Cu immiscible bimetallic composite. J. Alloys Compd..

[26] Han L., Liu J., Tang H., Yan Z. (2022). Study of Zr addition on the composition, crystallite size, microstructure and properties of high-performance nano Cu alloys prepared by mechanical alloying. Mater. Chem. Phys..

[27] Deng S., Li J., Hong N., Lu D.-D., Zeng G.-J., Liu Z., Xiang H., Ji-rong Q., Liu D. Effect of Zr addition on the microstructure evolution and mechanical properties of extruded Al-Cu-Li-Mn alloys. Mater. Charact..

[28] Bian X., Wang A., Xie J., Liu P., Mao Z., Chen Y., Liu Z., Gao Y. (2023). Atomic-scale deformation mechanisms of nano-polycrystalline Cu/Al layered composites: A molecular dynamics simulation. J. Mater. Res. Technol..

[29] Bian X., Wang A., Xie J., Liu P., Mao Z.P., Liu Z. (2023). Effect of Al_2_Cu constituent layer thickness discrepancy on the tensile mechanical behavior of Cu/Al_2_Cu/Al layered composites: A molecular dynamics simulation. Nanotechnology.

[30] Cai J., Ye Y.Y. (1996). Simple analytical embedded-atom-potential model including a long-range force for fcc metals and their alloys. Phys. Rev..

[31] Stukowski A. Structure identification methods for atomistic simulations of crystalline materials. Model. Simul. Mater. Sci. Eng..

[32] Wolde P.R.T., Ruiz-Montero M.J., Frenkel D. (1995). Numerical Evidence for bcc Ordering at the Surface of a Critical fcc Nucleus. Phys. Rev. Lett..

[33] Alizadeh J., Panjepour M., Ahmadian M. (2020). Modeling the Stretch Behavior of the Single-Crystal Ni–Al Alloy and Its Molecular Dynamics Simulation. Phys. Solid State.

[34] Wang J.R.G., Hoagland R.G., Hirth J.P., Misra A. (2008). Atomistic modeling of the interaction of glide dislocations with “weak” interfaces. Acta Mater..

[35] Niu J.J.Y., Zhang G., Liu P., Zhang P., Sun L., Zhang G.J., Sun J. (2012). Size-dependent deformation mechanisms and strain-rate sensitivity in nanostructured Cu/X (X = Cr, Zr) multilayer films. Acta Mater..

[36] Wang Y.Q., Zhang J.Y., Liang X., Wu K., Liu G., Sun J. (2015). Size- and constituent-dependent deformation mechanisms and strain rate sensitivity in nanolaminated crystalline Cu/amorphous Cu–Zr films. Acta Mater..

[37] Xiong Y., Hu W., Shu Y., Luo X., Zhang Z., He J., Yin C., Zheng K. (2022). Atomistic studies of the responses of composites with thermal residual stresses and defects under uniaxial loading. J. Alloys Compd..

[38] Li G., Wang Q., Shang Z., Luo L., Yan B., Jiang H., Ding W. (2019). An Investigation on Microstructures and Mechanical Properties of Ultra-Low Cu Layer Thickness Ratio Cu/8011/1060 Clads. Metall. Mater. Trans..

